# Different ossification patterns of intermuscular bones in fish with different swimming modes

**DOI:** 10.1242/bio.012856

**Published:** 2015-11-24

**Authors:** Wenjie Yao, Yaoping Lv, Xiaoling Gong, Jiaming Wu, Baolong Bao

**Affiliations:** 1 The Key Laboratory of Exploration and Utilization of Aquatic Genetic Resources, Shanghai Ocean University, Ministry of Education, Shanghai 201306, China; 2 College of Ecology, Lishui University, Lishui 323000, China

**Keywords:** Intermuscular bone, Ossification, *Anguilla japonica*, *Danio rerio*, Tail amputation

## Abstract

Intermuscular bones are found in the myosepta in teleosts. However, there is very little information on the development and ossification of these intermuscular bones. In this study, we performed an in-depth investigation of the ossification process during development in zebrafish (*Danio rerio*) and Japanese eel (*Anguilla japonica*). In Japanese eel, a typical anguilliform swimmer, the intermuscular bones ossified predominantly from the anterior to the posterior. By contrast, in the zebrafish, a sub-carangiform or carangiform swimmer, the intermuscular bones ossified predominantly from the posterior to the anterior regions of the fish. Furthermore, tail amputation affected the ossification of the intermuscular bones. The length of the intermuscular bones in the posterior area became significantly shorter in tail-amputated zebrafish and Japanese eels, and both had less active and lower swimming speeds; this indicates that swimming might induce the ossification of the intermuscular bones. Moreover, when a greater length of tail was amputated in the zebrafish, the intermuscular bones became even shorter. Tail amputation affected the length and ossification of intermuscular bones in the anterior part of the fish, close to the head, differently between the two fish: they became significantly shorter in the zebrafish, but did not in the Japanese eel. This might be because tail amputation did not significantly affect the undulations in the anterior of the Japanese eel, especially near the head. This study shows that the ossification of intermuscular bones might be induced through mechanical force loadings that are produced by swimming.

## Abstract (precis)

**Summary:** The ossification of intermuscular bones in teleost fish appears to be induced by mechanical force loadings produced by swimming.

## INTRODUCTION

Consecutive muscle segments are separated by myosepta with tendons inside. Some or all of these tendons can ossify along parts of the body (Gemballa et al., 2003) and are thought to be homologous with intermuscular bones in a number of teleosts (Danos and Ward, 2012). In reference to the site of attachment, intermuscular bones are classified into epineurals, epicentrals, and epipleurals, which attach via ligaments to neural, central, and hemal arches or ribs, respectively (Owen, 1846, 1866; Patterson and Johnson, 1995; Gemballa and Britz, 1998).

**List of abbreviations**

SL: standard length
BCF: body-caudal fin
PFA: paraformaldehyde
PBS: phosphate buffer saline
KOH: potassium hydroxide
ep: epipleurals
en: epineurals
ec: epicentrals
IB: intermuscular bone

Since intermuscular bone is ossified from tendons, its function was considered to be similar to that of the tendon. The functions of myoseptal tendons are believed to be: transmitting force between muscle segments (Westneat et al., 1993; Long et al., 2002; Donley et al., 2004), increasing overall body stiffness (Long and Nipper, 1996), and constraining myomere deformation during contraction (Azizi et al., 2002; Brainerd and Azizi, 2005). In accordance, an ossified rod, with the same shape as that of a collagenous tendon, should better resist forces in all directions. It has been supposed that the function of intermuscular bones in the carp, *Cyprinus carpio*, has been to constrain myomere deformation during contraction, and increase body stiffness (Ping, 1962; Organ, 2006). In addition, it is important that the presence, or absence, of tendon ossification within teleost fish was found to be correlated with morphological metrics of the fish, such as the position of the median fins, body aspect ratio and vertebral number, which in turn affect the distribution of forces experienced by the fish's body during locomotion (Danos and Ward, 2012).

Biomechanics may be a strong factor behind tendon ossification (Danos and Ward, 2012). Therefore, in order to better understand tendon ossification, it would be beneficial to compare ossification patterns of intermuscular bones in different types of swimmers. Intermuscular bones are thought to be formed through membranous ossification (Bird and Mabee, 2003). However, there is very little information on the development and ossification of intermuscular bones. In this study, we compared the ossification process of intermuscular bones, between Japanese eel (*Anguilla japonica*), a typical anguilliform swimmer, and zebrafish (*Danio rerio*), a sub-carangiform or carangiform swimmer. Furthermore, the ossification of intermuscular bones in the tails of amputated zebrafish and Japanese eel was investigated.

## RESULTS

### Intermuscular bones ossify predominantly from the posterior to the anterior, during development in zebrafish

**Fig. 1.**
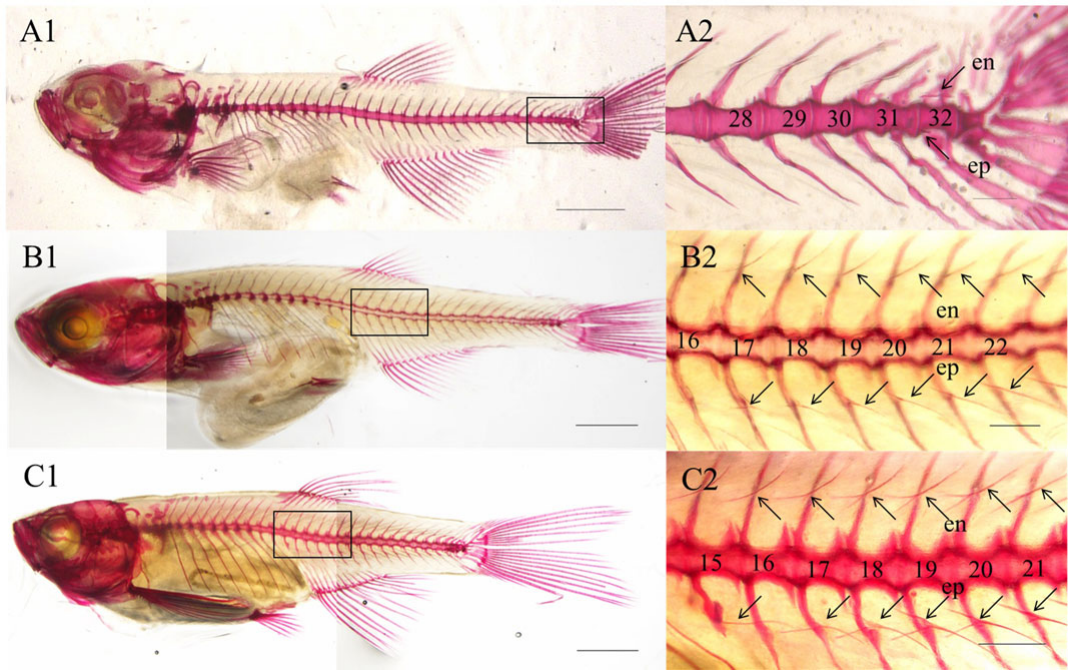
**Distribution and morphological specification of intermuscular bones during the development of the zebrafish.** (A1,B1,C1) The entire skeletons of three zebrafish specimens. Scale bar=500 μm. (A2,B2,C2) enlargement of the rectangular area in A1,B1,C1 showing intermuscular bones. Scale bar=200 μm. en, epineurals; ep, epipleurals.

In zebrafish, epineural bones are located in each myoseptum, while epipleural bones are only found in the myosepta within the tail area. Epicentral bones are not found in zebrafish (Table S1). Ossification of the intermuscular bones happened in the post embryo stages. The intermuscular bones began to ossify in the tail region of zebrafish that had grown to 6.52–7.63 mm standard length (SL) (Fig. 1A). Specifically, ossified epineural bones and epipleural bones were observed at approximately the 32nd vertebra (Fig. 1A2). Beginning at approximately the precaudal–caudal transition, the morphology of the epipleural bones was of a simple linear shape (type I) (Fig. 1B); however, Y-shaped ossified intermuscular bones were observed in zebrafish that were longer than 9.13 mm SL (Fig. 1C). With further development of the zebrafish, the ossified intermuscular bones were observed in the myosepta close to the head, and in 13.73 mm SL zebrafish, all of the intermuscular bones were ossified.

### The impacts of tail amputation on the ossification of intermuscular bones and swimming behavior in zebrafish

**Fig. 2.**
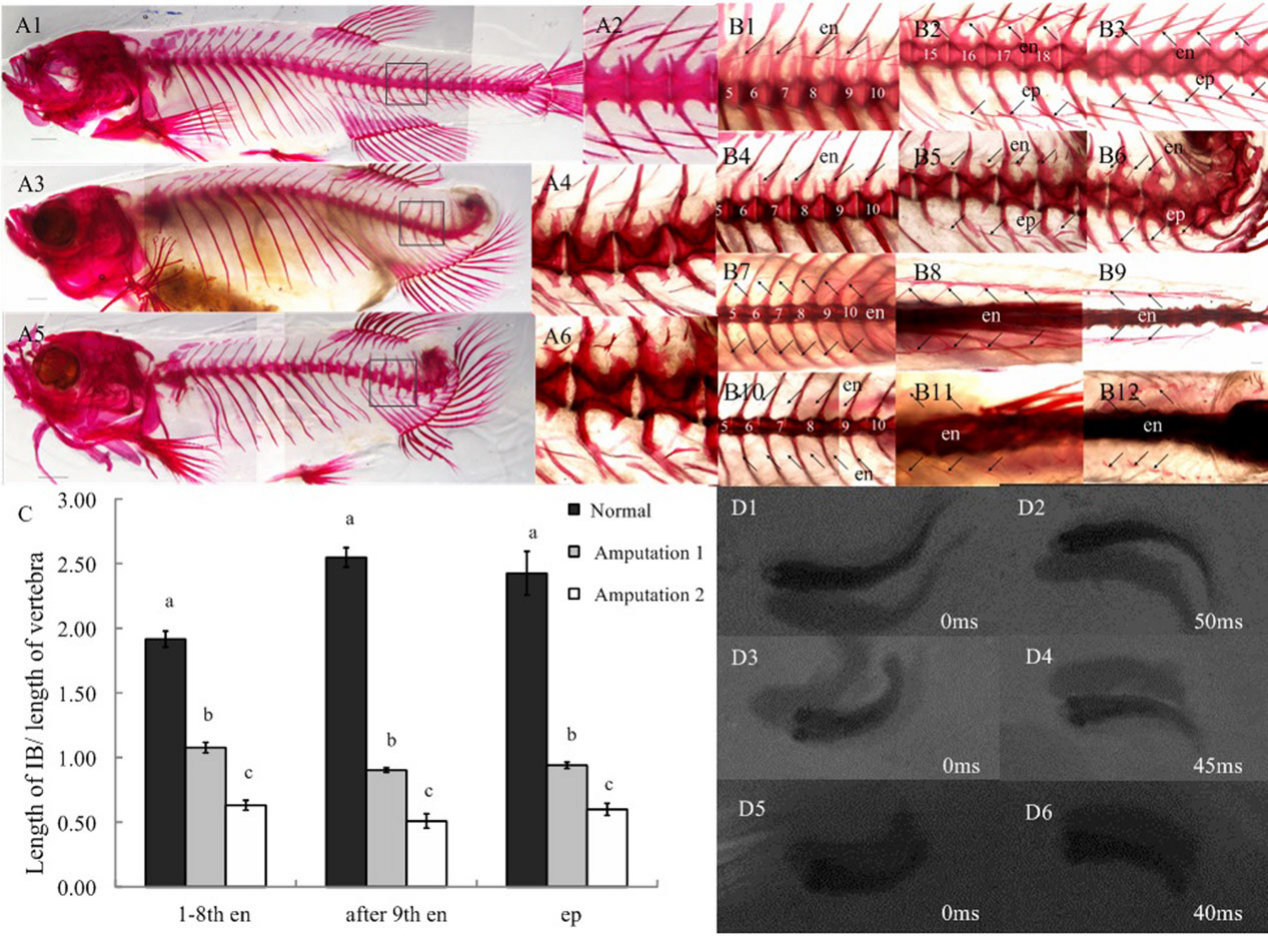
**The impact of tail amputation on the ossification of intermuscular bones and swimming behavior in the zebrafish.** (A) Whole-mount stained skeletons of the normal (A1) and tail-amputated (A3, Amputation 1; A5, Amputation 2) zebrafish. A2, A4 and A6 are enlarged views of A1, A3 and A5, respectively. Scale bar=500 μm. (B) The intermuscular bone stains from the lateral view (B1-B6) and the dorsal view (B7-B12) of normal (B1-B3 and B7-B9) and the tail-amputated (B4-B6 and B10-B12) zebrafish. Scale bar=500 μm. (C) The effects of amputation on the length of the intermuscular bone in zebrafish (means±s.d., different letters above the bars represent significant differences, *P*<0.01). (D) An example of a burst swim in normal (D1,D2) and tail-amputated (Amputation 1, D3,D4; Amputation 2: D5,D6) zebrafish. en, epineurals; ep, epipleurals.

Based on the above results, the question arose as to why the intermuscular bones began to ossify in the tail region? It was hypothesized that either some unknown morphogens could be secreted by cells or organs in the tail, or tail oscillation, could induce ossification of the intermuscular bones. Two kinds of tail amputation for zebrafish were conducted in this study in order to test the above hypotheses: in the first, tail amputation was performed just after the anal fin (at the 22nd–23rd vertebra), in the second, half of the caudal peduncle (at the 24th–26th vertebra) was removed from the zebrafish, 14 days after hatching. In both experiments, the intermuscular bones were still ossified, when analyzed 9 months after tail amputation (Fig. 2A), indicating that no morphogen is secreted in the tail area that induces ossification of the intermuscular bones.

However, both kinds of tail amputation did impact the ossification of the intermuscular bones. For example, the intermuscular bones between the 5th and 8th vertebrae became shorter (Fig. 2B1,B4) and the bending section disappeared in the dorsal view of the tails of amputated fishes (Fig. 2B7,B10). The normal shape of the intermuscular bones between the 15th and 18th vertebrae was Y-like, whereas at the same site in tail-amputated fishes, it became I-like or V-like (Fig. 2B2,B5). In the dorsal view, the forked part of the bones disappeared, so they looked the same as I type bones in the anterior of the body (Fig. 2B8,B11). The tail amputations also severely affected ossification of the intermuscular bones in the posterior area, where they became significantly shorter (Fig. 2B3,B6) or looked like dots (Fig. 2B12). Further statistical analysis showed that tail amputation significantly impaired the ossification of the intermuscular bones. The length of the intermuscular bones versus the length of vertebrae, between tail-amputated and normal zebrafish was also compared; after amputation, the length of all intermuscular bones became significantly shorter (*P*<0.01). There was a positive correlation between the length of the tail amputated and the shortening of the intermuscular bones (Fig. 2C; *P*<0.01), indicating that the ossification of the intermuscular bones might be induced by tail oscillation.

The tail-amputated zebrafish could still swim, but at a relatively slow speed. The longer the tail amputation, the slower their swim-speed became. Because the tail-amputated zebrafish most often performed a slow swim, rather than a burst swim, the normal fish exhibited fewer slow swims than the tail-amputated ones. According to the records of their locomotion, by high-speed camera, the tail-beat frequency of the tail-amputated zebrafish (approximately one cycle in 80/90 ms; 12.63±0.45/11.11±0.45 Hz) was high, relative to the tail-beat frequency of normal zebrafish (approximately one cycle in 100 ms; 9.62±0.33 Hz) during cyclic swimming (Fig. 2D). The stride length of the tail-amputated zebrafish (3.35±0.25/3.85±0.35 mm) was shorter, relative to the normal zebrafish (4.65±0.45 mm), during cyclic swimming (Table 1).

**Table 1.**
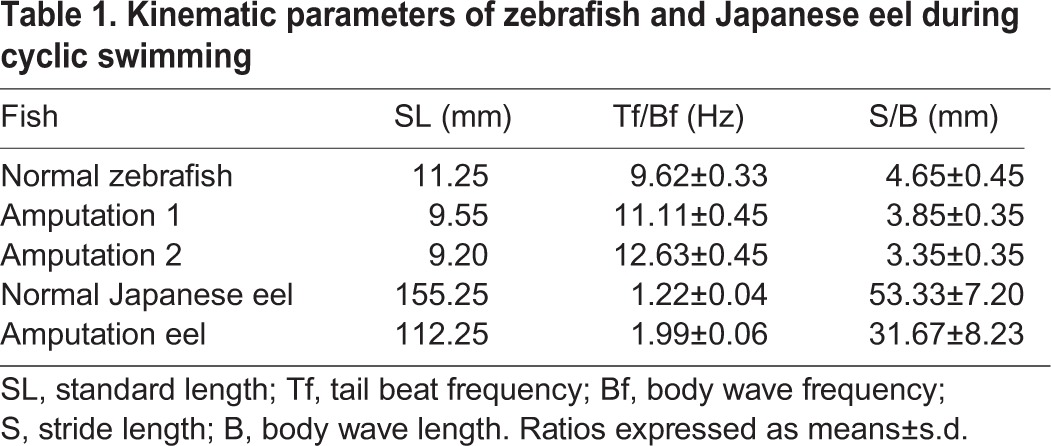
**Kinematic parameters of zebrafish and Japanese eel during cyclic swimming**

### Intermuscular bones ossify predominantly from the anterior to the posterior, during development in the Japanese eel

**Fig. 3.**
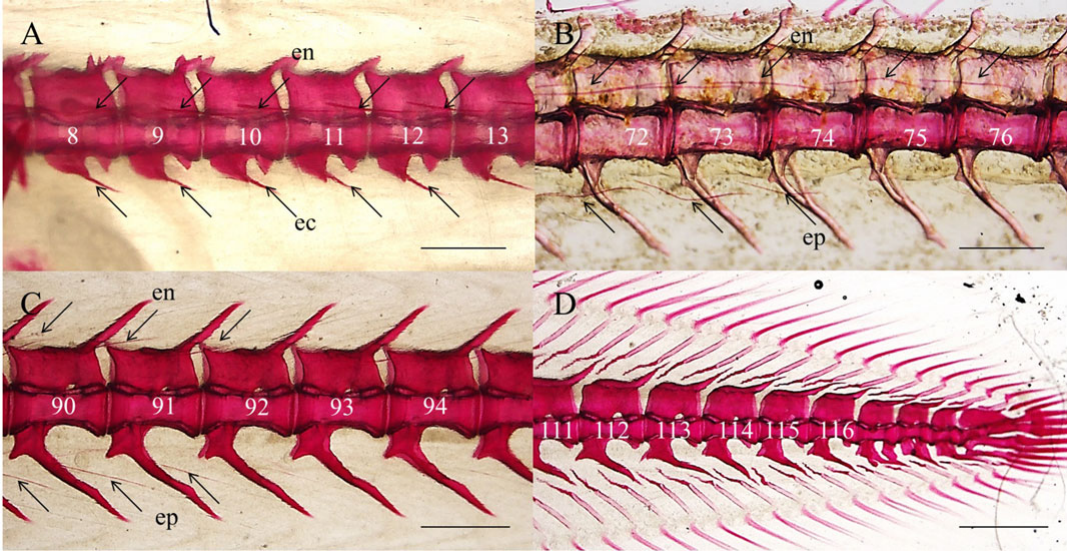
**Distribution and morphological specification of intermuscular bones in elvers of the Japanese eel.** (A) Whole-mount stained skeleton of the anterior region (near the head) in elvers. (B) Whole-mount stained skeleton of the posterior region of the Japanese eel. (C) Whole-mount stained skeleton of the region near the tail of the Japanese eel. (D) Whole-mount stained skeleton of the tail region of the Japanese eel; the intermuscular bones were still not ossified. Scale bar=500 μm. en, epineurals; ep, epipleurals; ec, epicentrals.

In adult Japanese eel, the epineural bones were located in each myoseptum, epicentral bones were located in the myosepta within the anterior region, and epipleural bones were only found in myosepta within the posterior area (Table S2). Similar to zebrafish, ossification of the intermuscular bones happened in the post embryo stages in the Japanese eel. Ossification of the epineural and epicentral bones located in the anterior region was observed in elvers (Fig. 3A). With growth, the epineural and epipleural bones located in the posterior region of the elvers ossified (Fig. 3B,C). In 176.79–221.44 mm SL Japanese eels, all intermuscular bones were ossified.

### Tail amputation impaired the ossification of intermuscular bones in the Japanese eel

**Fig. 4.**
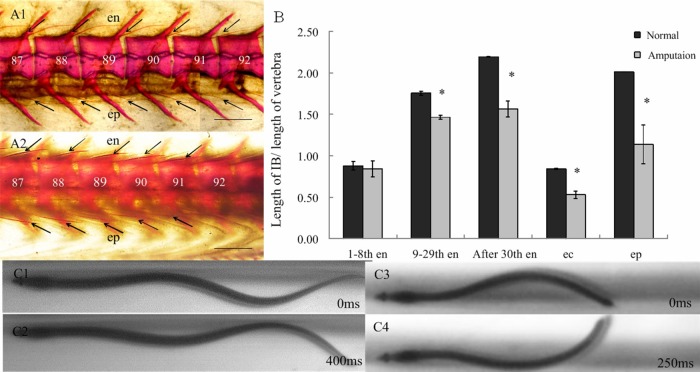
**The impact of tail amputation on the ossification of intermuscular bones and swimming behavior in the Japanese eel.** (A) Whole-mount stained skeleton of Japanese eel specimens after amputation (A1) and with no amputation (A2). Scale bar=500 μm. en, epineurals; ep, epipleurals. (B) Amputation has a no effect on length of intermuscular bones near the head, but has significant effect on the length of intermuscular bones in the posterior regions of the Japanese eel. Means±s.d., *P<0.05. (C) An example of the half body wave in normal (C1,C2) and tail-amputated (C3,C4) Japanese eel. en, epineurals; ep, epipleurals; ec, epicentrals.

Similar to the zebrafish, the tail-amputated Japanese eel also had ossified intermuscular bones, but they were also shorter than those in the normal eels (Fig. 4A). Further statistical analysis showed that the average length of the 1st–8th epineural bones did not significantly change. However, beyond the 9th epineural bone, the epineural bones, all the epicentral bones and the epipleural bones were significantly shorter (*P*<0.05) (Fig. 4B).

The tail-amputated Japanese eel could still swim, but at a relatively slow speed and stopped swimming more often, than normal eels. According to the records of their locomotion, by high-speed camera, the body wave frequency of the normal Japanese eels (approximately one cycle in 800 ms, 1.22±0.04 Hz) was relatively lower than the body wave frequency of the tail-amputated Japanese eels (approximately one cycle in 500 ms, 1.99±0.06 Hz; Fig. 4C). The stride length of the tail-amputated Japanese eels (31.67±8.23 mm) was also shorter, relative to that of the normal Japanese eels (53.33±7.20 mm), during cyclic swimming (Table 1).

## DISCUSSION

The ossification pattern of intermuscular bones during fish development has only been described in a few cases. Bird and Mabee (2003) previously observed that epineural bones developed and ossified predominantly from the posterior to the anterior regions in zebrafish. In this study, the more detailed investigation of the ossification sequences in zebrafish, and analysis of the morphological development of the intermuscular bones, provides further support to Bird and Mabee's (2003) study. Recently, the pattern of ossification of intermuscular bones, from the posterior to the anterior regions has also been shown in other Cyprinidae fishes, such as *Hypophthalmichthys molitrix*, *Hemibarbus labeo* and *Cyprinus carpio* var. *color* (Ke et al., 2008; Lv et al., 2012, 2014). However, in this study we found a different ossification pattern of the intermuscular bones, being predominantly from the anterior to the posterior regions, during the development of Japanese eels (*A. japonica*); this indicates that there exist different ossification patterns in different teleosts.

The different ossification patterns observed might be associated with different swimming modes between the fishes. Most fish generate thrust by bending their bodies into a backward-moving propulsive wave that extends to the fish's caudal fin; this type of swimming is classified as body and/or caudal fin (BCF) locomotion (Breder, 1926; Sfakiotakis et al., 1999). Like other species of eels, the Japanese eel is a typical anguilliform swimmer (within the BCF propulsion class). Most of the body of the Japanese eel participates in large amplitude undulations at high swimming speeds and during acceleration; at lower speeds, undulation is confined to the posterior region (Lauder and Tytell, 2005). Initial ossification of intermuscular membranes in the anterior part of the body in elvers, might improve the muscle's ability to generate a stronger thrust.

In contrast to the Japanese eel, the zebrafish is a sub-carangiform or carangiform swimmer (within the BCF propulsion class). The amplitude of the undulations in the zebrafish is limited in the anterior part of the body, and increases in the posterior half or the last third of the body (Plaut, 2000; Muller et al., 2008). Larval zebrafish often exhibit a relatively slow-speed pattern of swimming, characterized by small bend angles and a locus of maximal bending located near the tail of the fish (Budick and O'malley, 2000). Thus it is clear that in zebrafish the thrust generated is mainly reliant on the posterior part of the body. Body stiffness is important for propulsion by undulations (Long and Nipper, 1996); by starting ossification of the intermuscular tendons in the posterior region, it increases the stiffness of that body part and should help the larval zebrafish generate better thrust. Ossified myoseptal tendons may contribute to passive body flexural stiffness in knifefishes, of the order Gymnotiformes (e.g. *Apteronotus albifrons*), and of the distant family Notopteridae (e.g. *Notopterus chitala*) (Danos and Ward, 2012). Flexural stiffness has also been shown to be a significant mechanical property of undulating bodies that can dramatically alter the kinematics (Ping, 1962; Tytell, 2004).

Tail amputation affects the length of intermuscular bones differently in zebrafish and Japanese eel. In tail-amputated zebrafish, the ossification of all the intermuscular bones were impaired, especially in the posterior area; no matter where the intermuscular bone was, the length of the epineural and epipleural bones became significantly shorter. Moreover, if a greater length of tail was amputated, the intermuscular bones became even shorter. In contrast, in the tail-amputated eels, the intermuscular bones in the anterior part of body (1st–8th epineurals) did not become significantly shorter. The difference between the tail-amputated zebrafish and eel may be a result of their different swimming modes.

Tail amputation could also impair the fish swimming behavior. In this study, tail-amputated zebrafish and Japanese eel were less active and had lower swimming speeds than the normal fish, similar to observations in a previous study (Plaut, 2000), in which no-tail fish mutants were significantly less active than the wild type fish. However, in this study, the different types of swimmers (zebrafish versus Japanese eel) were affected by tail amputation slightly differently. The tail-amputated zebrafish took less time to accomplish a tail beat than normal individuals; for zebrafish, with a shorter tail the thrust produced by every tail-beat is smaller, so it would be expected that their muscular systems will be subjected to less mechanical force loadings induced by swimming. For the eels, the undulations in the posterior of the body were also affected by the tail amputations. It would be expected that the propulsive force would therefore be smaller, and their muscular systems will be subjected to less mechanical force loadings induced by swimming. However, in the anterior part of the Japanese eel, especially near the head, the undulations were nearly not affected. Indeed, the mechanical forces at play are thought to be important for the occurrence and evolution of the skeleton (Danos and Ward, 2012). External mechanical forces can regulate genetic pathways of both cartilage and bone development (Andreassen et al., 2001; Fan et al., 2006; Haasper et al., 2008; Robling et al., 2008; Wu et al., 2001; Sun et al., 2015).

Taken together, the ossification of intermuscular bones might have a relationship with mechanical force loadings that are induced by swimming.

## MATERIALS AND METHODS

### Fish maintenance

Wild-type zebrafish (*D. rerio*) were derived from the AB line, maintained in our laboratory. The fish were maintained at 28.5°C on a 14:10 h (light:dark) cycle. Embryos were developed in fish water, within an incubator maintained at 28.5°C, throughout the experiment. Japanese eels (*A. japonica*), at the glass eels stage, were collected from the Luchaogang estuary of the Yangtze River, using a stationary fishing net, and then acclimated gradually to fresh water in an indoor glass aquarium (28 length×22 width×30 height cm). The eels were provided shelter in PVC pipes (10 cm length×5 cm in diameter) and fed a diet of freeze-dried bloodworms, two times a day. Water temperatures in the aquarium were 19–21°C during the experiment. The adult Japanese eels were collected from Jiuduansha delta estuary of Yangtze River, using a stationary fishing net. All animal experimental protocols were approved by the Institutional Animal Care and Use Committee of Shanghai Ocean University.

### Anatomy and morphology of the intermuscular bones in the adult Japanese eels

The eels were anesthetized in 200 mg/l tricaine methanesulfonate solution (Guoyao, Shanghai, China). After measurements (standard length) were taken of the 3 sampled fish, each individual was parceled in gauze and boiled. The gauze was removed when the fish had cooled and the skin was carefully peeled off using tweezers. The intermuscular bones were picked, one by one, from each myoseptum and their morphological features were documented.

### Amputation of the fish tails

The samples of zebrafish (14 days after hatching, 4.21±0.4 mm) were anesthetized in 200 mg/l tricaine methanesulfonate solution (Guoyao, Shanghai, China), before the amputation process. The tails of 60 fish were amputated using a sterilized razor blade. Two kinds of amputation were performed, for one, half of the caudal peduncle was cut off (at the 24th–26th vertebra), and for the other, the amputation site was just after the anal fin (at the 22nd–23th vertebra). The cut parts were immediately cleaned with 70% alcohol and soaked in Betadine microbicidal solution (Guoyao). Ten of the amputated fishes were subsequently transferred into each of three properly cleaned and disinfected glass aquaria (28 length×22 width×30 height cm). Each pool was maintained with 15 litre of water at 28.5°C throughout the experimental period of 9 months.

The amputation process of the glass eels' tails was conducted in the same way as for the zebrafish. The amputation site was two-thirds of the way along the body, which is about at the 68th-78th vertebra. In total, 15 of the amputated fish were transferred to a glass aquarium (28 length×22 width×30 height cm) and maintained at 19–21°C, for 11 months.

### Alizarin Red staining

Before fixing with 4% paraformaldehyde (PFA) at room temperature to stain the bones, both the normal and tail-amputated fishes were anesthetized with 200 mg/l of tricaine methanesulfonate solution (Guoyao). Alizarin Red staining was performed to visualize the intermuscular bones, according to a published protocol (Walker and Kimmel, 2007). In brief, after being washed twice with phosphate buffer saline (PBS), to remove the 4% PFA, the samples were washed in TBST (50 mmol/l Tris at pH 7.4, 150 mmol/l NaCl, 0.1% [v/v] Triton X-100), for 30 min. They were then placed in 1% trypsin for digestion and transferred to 2 mg/ml Alizarin Red in 1% potassium hydroxide (KOH) for 1 h, before finally being cleared in 20% glycerol and 1% KOH for 40 min. Alizarin Red-stained samples were photographed and preserved in a solution containing four parts 100% glycerol plus one part 95% ethanol (v/v). Samples were measured, observed and photographed using a dissecting microscope (SMZ1500, Nikon, Tokyo, Japan), and photographs were processed using NIS-Element D software (Nikon).

### Swimming behavior

To observe the swimming behavior of the fish, each fish was transferred into a plastic tank (37 length×24 width×11 height cm) containing chlorine free bore-well water. The tanks were filled to a depth of approximately 4 cm. The behavior of each fish was captured with a MD4256 digital camera (Nikon), attached to a high-speed camera system (IDT Y3-S2 Edition, Italy) running on a PC. All data were collected at 1000 frames/s and 20 swimming sequences for each fish were recorded.

### Statistical analysis

Significant differences between the lengths of the intermuscular bones of tail-amputated and normal fish were tested using a nonparametric test (Wilcoxon signed-ranked Test). SPSS 19.0 (SPSS Inc., Chicago, USA) was used for the statistical analysis. A difference was considered significant if *P*<0.05. Data are reported as mean±s.d.

Five sequences out of the 20 recorded sequences of swimming behavior were selected for analysis. Each analyzed sequence consisted of at least two complete tail beats. For each sequence of cyclic swimming behavior from each fish, 10 complete tail beats or body waves were recorded. Tail beat frequency is reciprocal to the time it takes the tail to complete one tail beat, and the same is true for the body wave frequency. The stride length (distance covered during one tail beat) and body wave length (distance covered during one body wave) were also determined. Significant differences between the lengths of the intermuscular bones in the tail-amputated and normal fish were tested using a nonparametric test (Wilcoxon signed-ranked test). As above, SPSS 19.0 (SPSS Inc.) was used for the statistical analysis and the difference was considered significant if *P*<0.05. Data reported are mean±s.d.
